# Editorial: Opportunities and challenges of human preconception research

**DOI:** 10.3389/frph.2024.1508151

**Published:** 2024-11-27

**Authors:** Linda G. Kahn, Evelyn Loo, Gita D. Mishra, Joseph B. Stanford

**Affiliations:** ^1^ Departments of Pediatrics and Population Health, New York University Grossman School of Medicine, New York, NY, United States; ^2^ Institute of Human Development and Potential (A*STAR) and Department of Paediatrics, Yong Loo Lin School of Medicine, National University of Singapore, Singapore, Singapore; ^3^ Australian Women and Girls' Health Research Centre, School of Public Health, University of Queensland, Herston, QLD, Australia; ^4^ Department of Family and Preventive Medicine, University of Utah, Salt Lake City, UT, United States

**Keywords:** preconception, time to pregnancy, study design, epidemiology, remote biospecimen collection, menstrual cycle tracking apps, life course

**Editorial on the Research Topic**
Opportunities and challenges of human preconception research

Accumulating evidence suggests that pre- and periconception time periods are critical windows in which acute or cumulative exposure to environmental factors, including neighborhood, behavioral, psychosocial, dietary, and chemical, may influence gamete and embryo development in ways that affect fetal and child health. Preconception health and environmental exposures have also been associated with predictors of male (1, 2) and female (3–6) fecundity (e.g., semen and oocyte quality, gynecologic conditions such as polycystic ovarian syndrome and endometriosis) that, in turn, have been linked to long-term morbidity and mortality in men (7, 8) and women (9, 10). Preconception studies, therefore, may shed light on the earliest precursors of disease and have the potential to contribute to our understanding of health across the life course as well as the intergenerational transmission of health. The four articles contained in this Research Topic address various aspects of human preconception research, including study design, study results, and translation of knowledge to clinical and public health practice.

Preconception studies are notoriously difficult to conduct and pose distinct challenges and limitations. While studies that recruit individuals or couples trying to conceive benefit from having motivated participants, they result in highly selected cohorts, yielding results that may not be generalizable. Pregnancy planners differ from the general population along sociodemographic axes, including age, race/ethnicity, income, and education (11, 12). Studies focused on assessing time to pregnancy, a measure of couple fecundity, restrict to those who are trying to conceive naturally through unprotected heterosexual intercourse, which excludes not only those receiving ovarian stimulation, intrauterine insemination or *in vitro* fertilization, but also single women and same-sex couples. Conversely, studies focused on data and biospecimen collection to assess preconception exposures in relation to gamete and/or embryo development often recruit from fertility clinics, resulting in a highly selected sample and risking confounding by indication. Preconception analyses are often conducted using retrospective data collected from birth cohort participants, however these results are generalizable only to those successfully able to conceive. Furthermore, when it is time to analyze results, researchers must account for biases of selection, survival, or enhanced surveillance for outcomes related to conception, pregnancy, live birth, and child health.

The largest preconception study to date is the ongoing Pregnancy Study Online (PRESTO), a North American web-based preconception cohort study that recruits female-identifying participants age 21–45 years from the United States and Canada who are actively attempting pregnancy through heterosexual intercourse without medical intervention, then encourages them to invite their male partners to enroll, as well (13). While it aims to examine how the preconception environment influences reproductive outcomes, it has heretofore been limited in its ability to assess health biomarkers or chemical exposures because of the infeasibility of collecting biological samples from participants outside of two metropolitan areas where they had study sites. The researchers recently piloted remote biospecimen collection, in which they asked participants to mail in urine and blood samples, and in this issue compare the in-person and mail-based approaches in terms of the protocol design, the demographics of those who consented to participate in each protocol, and the costs per sample collected. Koenig et al. provide a detailed accounting of their methods and frank discussion of the challenges they encountered that will be immensely helpful to those contemplating remote biospecimen collection in any context, not only preconception research.

Another approach to collecting preconception data at scale that has garnered much interest is by leveraging commercial menstrual cycle tracking apps. Jukic et al. report results of a pilot study designed to characterize app users with the goal of understanding the underlying demographics of the population in anticipation of conducting a larger time-to-pregnancy study that will use app-based recruitment. They partnered with Ovia Fertility, a free menstrual cycle tracking app, and sent an email to a random sample of users age ≥18 years in the United States with a link to an online survey that collected demographic data as well as information about their pregnancy status and intention, reproductive and general health history, and height and weight. As with PRESTO, respondents were asked to invite their partners to participate, in this case by answering questions at the end of the survey. In addition to quantifying the potential recruitment yield for their future time-to-pregnancy study, the authors provide valuable information on aspects of user health and behavior that underscore the potential of menstruation cycle tracking apps to study other aspects of preconception and reproductive health.

While the preconception period is generally perceived to refer to the months immediately preceding conception, because oocytes are all created prior to birth, exposures that affect oocyte quality and hence fetal and pregnancy health can occur at any time in the female life course prior to pregnancy. Hipwell et al. take such a life course approach in their analysis of stress exposure throughout childhood and adolescence in relation to birth outcomes. Their study is nested within the Pittsburgh Girls Study, an ongoing longitudinal cohort that enrolled girls age 5–8 years and oversampled from low-income neighborhoods (14). While the original aim of the study was to describe the co-occurrence of the development of behavior and mental health problems in girls, annual assessments over more than 20 years have provided an opportunity to examine a host of other outcomes, including pregnancy outcomes among those who have gone on to have children of their own. The repurposing of a pediatric cohort to provide preconception data for the next generation is an innovative approach that allows for investigation of a far broader range of exposures at multiple potentially critical periods of reproductive development that may be as relevant as—if not more so than—the months immediately preceding conception.

Discovering associations of preconception health with pregnancy and child outcomes is all very interesting from an epidemiologic point of view, but the fundamental purpose of epidemiologic research is to improve public health, so it is important that findings be disseminated to those who might directly benefit from them. How best to do so is the focus of Daly et al. who interviewed twenty women in the West of England about their receptivity to various methods for delivering preconception health advice and approaches to potential interventions. The themes that emerged around accessibility, discretion, and trustworthiness, as well as opinions as to desirable—and undesirable—content are relevant to clinicians, health educators, and anyone planning a campaign to improve preconception health.

Collectively, the studies in this special issue highlight the growing interest in preconception health research and posit some innovative options for study design, participant recruitment, and data collection, as well as communication of results to the target population. Despite the challenges of preconception research, there are many opportunities to understand and influence human health. These include the examination of biological mechanisms underlying reproductive development and gamete production, genetic and epigenetic factors that influence fecundity and fetal programming, as well as interactions between social and environmental exposures during critical life stages that affect reproductive health across the life course, and potentially across generations. Finally, while preconception studies have occasionally involved male partners, few have followed maternal-paternal-child triads longitudinally. The associations of male preconception health not only with semen quality, but also with pregnancy and offspring outcomes is an area ripe for future research.

## References

[1] Bonde JP Flachs EM Rimborg S Glazer CH Giwercman A Ramlau-Hansen CH The epidemiologic evidence linking prenatal and postnatal exposure to endocrine disrupting chemicals with male reproductive disorders: a systematic review and meta-analysis. Hum Reprod Update. (2016) 23(1):104–25. 10.1093/humupd/dmw036 27655588 PMC5155570

[2] Salas-Huetos A Bulló M Salas-Salvadó J. Dietary patterns, foods and nutrients in male fertility parameters and fecundability: a systematic review of observational studies. Hum Reprod Update. (2017) 23(4):371–89. 10.1093/humupd/dmx006 28333357

[3] Zhang Y Ma NY. Environmental risk factors for endometriosis: an Umbrella review of a meta-analysis of 354 observational studies with over 5 million populations. Front Med (Lausanne). (2021) 8:680833. 10.3389/fmed.2021.680833 34760897 PMC8573094

[4] Sirohi D Al Ramadhani R Knibbs LD. Environmental exposures to endocrine disrupting chemicals (EDCs) and their role in endometriosis: a systematic literature review. Rev Environ Health. (2021) 36(1):101–15. 10.1515/reveh-2020-0046 32903210

[5] Kazemi M Kim JY Wan C Xiong JD Michalak J Xavier IB Comparison of dietary and physical activity behaviors in women with and without polycystic ovary syndrome: a systematic review and meta-analysis of 39 471 women. Hum Reprod Update. (2022) 28(6):910–55. 10.1093/humupd/dmac023 35639552 PMC9629501

[6] Priya K Setty M Babu UV Pai KSR. Implications of environmental toxicants on ovarian follicles: how it can adversely affect the female fertility? Environ Sci Pollut Res Int. (2021) 28(48):67925–39. 10.1007/s11356-021-16489-4 34628616 PMC8718383

[7] Eisenberg ML Li S Behr B Pera RR Cullen MR. Relationship between semen production and medical comorbidity. Fertil Steril. (2015) 103(1):66–71. 10.1016/j.fertnstert.2014.10.017 25497466

[8] Eisenberg ML Li S Cullen MR Baker LC. Increased risk of incident chronic medical conditions in infertile men: analysis of United States claims data. Fertil Steril. (2016) 105(3):629–36. 10.1016/j.fertnstert.2015.11.011 26674559

[9] Farland LV Harris HR. Long-term health consequences of endometriosis - pathways and mediation by treatment. Curr Obstet Gynecol Rep. (2020) 9(3):79–88. 10.1007/s13669-020-00287-9 33552675 PMC7864355

[10] Anagnostis P Tarlatzis BC Kauffman RP. Polycystic ovarian syndrome (PCOS): long-term metabolic consequences. Metabolism. (2018) 86:33–43. 10.1016/j.metabol.2017.09.016 29024702

[11] Finer LB Zolna MR. Declines in unintended pregnancy in the United States, 2008–2011. N Engl J Med. (2016) 374(9):843–52. 10.1056/NEJMsa1506575 26962904 PMC4861155

[12] Dehlendorf C Rodriguez MI Levy K Borrero S Steinauer J. Disparities in family planning. Am J Obstet Gynecol. (2010) 202(3):214–20. 10.1016/j.ajog.2009.08.022 20207237 PMC2835625

[13] Wise LA Rothman KJ Mikkelsen EM Stanford JB Wesselink AK McKinnon C Design and conduct of an internet-based preconception cohort study in North America: pregnancy study online. Paediatr Perinat Epidemiol. (2015) 29(4):360–71. 10.1111/ppe.12201 26111445 PMC4662659

[14] Keenan K Hipwell A Chung T Stepp S Stouthamer-Loeber M Loeber R The Pittsburgh girls study: overview and initial findings. J Clin Child Adolesc Psychol. (2010) 39(4):506–21. 10.1080/15374416.2010.486320 20589562 PMC2946599

